# A New Natural Processing System Based on Slight Carbon Dioxide Pressure for Producing Black Table Olives with Low Salt Content

**DOI:** 10.3390/foods12213950

**Published:** 2023-10-29

**Authors:** Gino Ciafardini, Biagi Angelo Zullo

**Affiliations:** Department of Agricultural, Environmental and Food Sciences, University of Molise, Via de Sanctis, I-86100 Campobasso, Italy; ciafardi@libero.it

**Keywords:** debittering, low salt, natural processing, pressurized CO_2_, table olive

## Abstract

Naturally fermented black table olives are usually processed in brine with low pH and high NaCl content. Because salt is responsible for several cardiovascular problems, methods are needed to decrease the salt (NaCl) content in olive pulp. This study investigated a new natural processing system wherein microorganism growth is inhibited by slight pressure of CO_2_ (spCO_2_), in addition to low pH and NaCl, in brine with decreased salt content. The fermentation performed under spCO_2_ with a low-salt brine with 6% (*w v*^−1^) NaCl and 0.5% (*w v*^−1^) citric acid, unlike the traditional system, inhibited the growth of bacteria and fungi and decreased the concentration of yeasts. Processing tests with spCO_2_ in the presence of different salt and citric acid concentrations indicated a slight decrease in yeasts in brines containing 6% (*w v*^−1^) NaCl and 0.6% (*w v*^−1^) citric acid but not after inoculation of the same brines with *Saccharomyces cerevisiae.* In contrast, in the presence of 11% (*w v*^−1^) NaCl and 0.3% or 0.6% (*w v*^−1^) citric acid, the inhibitory effect of brines was greater compared to those with low-salt and it was also confirmed in the same brines inoculated with *S. cerevisiae.*

## 1. Introduction

Table olives are the oldest and most commonly used fermented vegetable product in the Mediterranean area, and they are also produced and consumed in countries worldwide, including the USA, Argentina, Peru, and Australia [1]. Freshly harvested olives cannot be consumed directly, because the olive pulp contains phenolic compounds, such as oleuropein and ligstroside, as well as aglycones, which impart a bitter taste to the fruit. To make olives edible, the bitter taste must be removed through the hydrolysis of oleuropein and its aglycones into simpler compounds with a pleasant flavor [2]. Table olives are prepared through three major methods: the Spanish process, in which green olives are treated with lye; the Californian process, which incorporates lye treatment and air oxidation; and the natural system, which involves spontaneous fermentation of the fruits in brines with differing salt content. In the natural processing system for black table olives, olives are directly stored for 8–12 months in brine without any prior pre-treatment [3]. The olives are typically rinsed with water, sorted according to the diameter of the fruit, and then placed in 160–200 L of polyethylene or polyvinyl chloride plastic barrels. The barrels are subsequently filled with freshly prepared brine containing 8–12% NaCl (*w v*^−1^), and, for safety reasons, lactic or citric acid [4,5]. Olives are fermented in the brine until they lose their bitter taste, and after 6 or more months, they are placed in jars with fresh brine and pasteurized. In this processing system, the removal of bitter compounds, primarily oleuropein and its aglycons, occurs via the enzymatic activity (β-glucosidase and esterase) of the fruits and the brine microbiota, as well as the diffusion of the phenolic compounds into the brine [6]. In the natural process for black table olives, several unresolved challenges are associated with the traditional methods used in each production area. One critical issue is the difficulty in maintaining the same anaerobic conditions necessary for correct fermentation in all barrels, each with its own ecosystem, to standardizing the characteristics of the final product. The microbial species diversity on the epicarp, the influence of the external temperature, the recycling of different types of empty barrels in subsequent processing cycles, and the infiltration of air into the barrels during top-up operations are sources of uncertainty affecting the characteristics of the finished product [7]. The penetration of oxygen into the barrels during top-up operations is a major problem, because it fosters the growth of undesirable oxidizing microorganisms that produce microbial biofilms on the top layer of the brine, thus causing quality defects. Recent studies have successfully controlled microbial biofilm formation on the brine during fermentation in barrels with an air-protected headspace [5]. To prevent spoilage due to the above factors, the traditional natural fermentation of black table olives uses high concentrations of NaCl salt. However, beyond the need to prevent spoilage defects, naturally processed black table olives could benefit from a decrease in NaCl content to meet the increased consumer demand for healthful products. Although the nutritional properties of table olives are well known, their frequent consumption may be hindered by their high NaCl content particularly that in naturally fermented black table olives, in which 8–12% (*w v*^−1^) NaCl is usually used to prevent spoilage. Reducing the NaCl concentration in the brine is strongly recommended because, as reported by the World Health Organization [8], a high salt intake of dietary sodium by consumers increases the risk of various diseases, such as hypertension and cardiovascular disease [9]. Mounting concerns regarding salt ingestion are reflected in recommended dietary intake values. The dietary guidelines for Americans indicate that salt should be limited to 2.3 g Na day^−1^ [10], and the EU reference intake has been set at 2.4 g Na day^−1^ [11]. Thus, decreasing the NaCl content in the brine during olive fermentation and in the final product has gained attention in the scientific community in the past decade [12]. Recent studies have evaluated the substitution of NaCl with KCl, although this alternative remains not universally accepted by olive producers or consumers, and requires knowledge of how to modify traditional methods [13,14]. As described above, both preventing brine microbial biofilm formation due to oxygen penetration into the barrels and decreasing the salt content are imperative. Because a lower salt content does not always ensure correct fermentation, to achieve these objectives, a third measure to prevent microbial growth during fermentation, in addition to pH and NaCl, must be included in the brine. Excessively decreasing NaCl in the brine of naturally processed black table olives additionally has detrimental effects on the texture, flavor, and shelf life of the final product, and the inhibition of microorganisms causing spoilage cannot be ensured. Therefore, decreasing the salt content in the brine of naturally fermented black olives without damaging the quality of the product remains a pressing problem. In a previous study, we performed the first investigation of the antimicrobial activity of brine treated with slightly pressurized CO_2_ (spCO_2_). This study provides a theoretical basis for increasing the antimicrobial activity of brine with low-salt content in naturally fermented black table olives [15]. The CO_2_ dissolved in the brine decreases the pH and simultaneously penetrates microbial cells and exerts growth inhibitory effects [16]. To slow the growth of aerobic bacteria and fungi, the food industry currently uses CO_2_ for modified-atmosphere packaging capable of extending the shelf life of various commodities under nonpressurized conditions [17,18]. High-pressure CO_2_, involving the use of pressurized CO_2_ between 50 MPa and 600 MPa, is applied to food at room temperature as a non-thermal pasteurization technology [19,20]. SpCO_2_ less than one megapascal (10 bar) may be a promising means of controlling microbial growth. Tests performed on a pilot plant scale have shown that spCO_2_ completely inhibits the growth of bacteria and fungi in brine with a decreased saline concentration [15]. However, beyond these pilot studies, spCO_2_ has never been used in black table olives naturally processed with a decreased salt content. To decrease the NaCl content in the debitterized olives, a new natural processing system for black table olives was studied herein, in which spCO_2_ was used in addition to pH and NaCl to prevent microbial growth.

## 2. Materials and Methods

### 2.1. Incubation Tests in Anaerobiosis and spCO_2_ Conditions

Experiments were performed on the fruit of the Leccino cultivar (*Olea europaea* L.). The olives were harvested at the ripening stage, corresponding to a 70% black surface color, from mid-September to mid-October during the 2021 season. After harvesting, the leaves and other inert materials were removed from olives, and the fruits were washed with tap water, sorted according to size, and placed in six 20 L polyethylene terephthalate (PET) demijohn bottles equipped with large self-sealing screw caps. Each demijohn bottle contained 15 kg olives covered with brine containing 6% (*w v*^−1^) NaCl and 0.5% (*w v*^−1^) citric acid. Finally, three demijohn bottles not hermetically closed with a cap were incubated under anaerobiosis conditions at atmospheric pressure, whereas other demijohn bottles were hermetically closed with a screw cap and incubated under spCO_2_ conditions. The incubation in anaerobiosis conditions occurred at atmospheric pressure, because the fermentation gases were expelled through the screw cap of the non-hermetic closure while the evaporated brine was replenished with fresh brine. In contrast, incubation under the spCO_2_ condition constantly occurred under 2 bar pressure with a valve device arising from the CO_2_ self-produced inside demijohn bottles by the respiratory activity of the fruits and the fermentation by the microbiota. The demijohn bottles with olives in brine were incubated for 12 months at 15–17 °C; after 1 month and every 3 months thereafter, brine samples were collected in axenic conditions through the screw cap and the valve device and subjected to the analysis described below.

#### 2.1.1. Microbiological Analysis and Yeast Biodiversity

Microbiological analysis was performed on the brine samples to enumerate the main microbial groups (total yeasts, total bacteria, and total molds) implicated in natural black table olive fermentation [21]. The brine samples were serially diluted by a factor of 10 in sterile Ringer’s solution (0.9% NaCl, *w v*^−1^), and the various dilutions were spread on specific agar media, as described below. Total yeast was evaluated on malt, yeast, glucose, peptone (MYGP) agar medium with the following composition, 3 g yeast extract (Biolife, Milan, Italy), 3 g malt extract (BBL, Cockeysville, MD, USA), 2.5 g casein bactotryptone (BD, Sparks, MD, USA), 2.5 g soy peptone (Biolife), 10 g D-glucose (Merck, Darmstadt, Germany), and 1000 mL distilled water, pH 7, supplemented with 100 µg mL^−1^ chloramphenicol (Sigma-Aldrich, Milan, Italy) to prevent the growth of bacteria, and incubated at 30 °C for 5 days. Total bacteria were enumerated after 24 h of incubation at 30 °C on nutrient agar (CM0003, Oxoid, Basingstoke, UK) supplemented with 0.05% (*w v*^−1^) cycloheximide (Sigma-Aldrich, Milan, Italy) to prevent yeast growth. The total mold was evaluated after 7 days of incubation at 28 °C on glucose yeast extract agar (GYEA; Oxoid, Basingstoke, UK). All plates were visually examined for typical colony types and morphological characteristics, which were recorded with the corresponding growth medium. The results are expressed as Log values of colony-forming units (CFU) per mL of brine (Log CFU mL^−1^). The detection limit was established as 10 CFU mL^−1^. Three replicates of each brine sample taken from each demijohn bottle were incubated under anaerobic and spCO_2_ conditions. Approximately 1000 yeast colonies isolated from each brine sample were used to set up master cultures in CHROMagar Candida medium (BBL, code 4354093, Heidelberg, Germany) [22]. On the basis of the chromogenic characteristics of the colonies, the presence of pseudohyphae, and the cell shapes and sizes, the yeasts were divided into homogeneous chromogenic groups. From each homogeneous chromogenic yeast group, ten isolates of the most frequently observed yeasts were chosen at random and identified at the species level by sequencing of the D1/D2 region (approximately 600 bp) of the large (26S) ribosomal subunit gene, as previously reported [15].

#### 2.1.2. Physicochemical Analysis

Physicochemical analysis was performed on both the brine and the fruits after 1 year of incubation under anaerobiosis and spCO_2_ conditions. The analyses involved determination of pH, titratable acidity, NaCl concentration, and total polar phenols content. The brine was directly analyzed without any pre-treatment, whereas the fruits were extracted as follows before analysis. The olives were first washed with distilled water, dried on paper, and pitted with a laboratory device; 10 g of olive pulp was placed in a beaker, and 50 mL of a methanol-distilled water (80:20, *v v*^−1^) mixture was added. After 2 min of homogenization (Turrax model T25; IKA, Milan, Italy), the paste was centrifuged 9000× *g* for 10 min using a Hettich centrifuge (Centrifuge model Universal 32, Hettich Instruments, Tüttlingen, Germany). Some parameters were evaluated in the supernatant in the same manner as the brine. The brine pH was assessed via a pH meter using an In Lab Routine Probe (Mettler, Toledo, OH, USA). The titratable acidity of the olive brine was determined via titration with 0.1 N NaOH solution. The equivalence point was determined through potentiometry at pH 8.3. The sodium chloride content of both the olive brine and olive pulp was assessed using the Mohr method: 1 mL of sample was first diluted with 50 mL of distilled water, and then titrated with 0.1 N AgNO_3_ by using K_2_CrO_4_ as the indicator [23]. The results are expressed as a percentage (*w v*^−1^). The total polar phenols content of both the brine and olive pulp was evaluated using the Folin–Ciocalteu colorimetric method, as described by Ciafardini and Zullo [5]. The K_225_ of the olive pulp was evaluated through dilution of 1 mL of sample with the same methanol–distilled water (80:20, *v v*^−1^) mixture adjusted to a final volume of 100 mL, and then read on a spectrophotometer (model 6300, Jenway, Essex, UK) at 225 nm. K_225_ is expressed as ABS_225_ g^−1^ olive pulp. Each chemical analysis was repeated three times.

#### 2.1.3. Sensory Analysis

The gustatory attributes related to the acid, salty, bitter, abnormal fermentation, musty, rancid and overall acceptability of Leccino black table olives naturally processed under anaerobiosis and spCO_2_ conditions after final bottling for 1 year were evaluated by trained evaluators (five men and five women aged 22–45 years of age) according to the International Olive Council (IOC) indications [24]. The sensory score attributes were as follows: unsatisfactory (1 point), moderate (2 points), good (3 points), and excellent (4 points). 

### 2.2. Table Olive Processing Tests with spCO_2_

The processing tests with spCO_2_ were performed on the same batch of Leccino olives described above, which had been marinated in brines with different concentrations of NaCl and titratable acidity, with or without enrichment with *Saccharomyces cerevisiae*. The olives, after being washed and classified according to size, were placed in 24 PET (5 L) demijohn bottles with self-sealing screw caps. The brine in 12 demijohn bottles was inoculated with a 0.1% suspension of 10^7^ CFU mL^−1^ of *S. cerevisiae* strain 2111 (DAEFS of Campobasso, Molise, Italy), whereas that in the remaining demijohn bottles did not undergo any microbial enrichment. Both types of brines described above contained 6% and 11% (*w v*^−1^) NaCl, respectively, with 0.3% (*w v*^−1^) and 0.6% (*w v*^−1^) citric acid. The low salt concentration was chosen in accordance with the IOC, which recommends storage with a minimum content of 6% (*w v*^−1^) NaCl in the brines of olives previously processed in the natural style [25]. The highest concentration of NaCl of 11% (*w v*^−1^) was chosen, because it is one of the most frequently used salt concentrations in black table olive processing, while the yeast *S. cerevisiae* was chosen considering the results of previous studies [15]. Each 5 L PET demijohn bottle was filled with 2.6 kg of olives and 2.4 L of brine. After filling, the demijohn bottles were hermetically closed to encourage the establishment of spCO_2_ inside them and incubated for 12 months at 15–17 °C. During the incubation, brine samples were collected every 3 months, in an axenic manner, through the action of spCO_2_ after slight loosening of the threaded caps. At the end of the incubation, both the brine and olive samples were analyzed. All samples were subjected to microbiological, physicochemical, and sensorial analyses using the methods described above. Each treatment was repeated three times.

#### Scanning Electron Microscope (SEM) Observation

Microorganisms suspended in the brine fraction were collected through centrifugation at 7000× *g* (Hettich Instruments) for 5 min. The microbial biomass was fixed in 1 mL of 3% (*v v*^−1^) glutaraldehyde (Sigma-Aldrich) in 0.1 M phosphate buffer, pH 7.2, for 12 h. The samples were rinsed twice in the same buffer and then dehydrated (twice for each solution) in a graded ethanol series (20, 40, 60, 80, 95, and 100%) for 10 min, with a final wash in acetone to achieve better CO_2_ substitution during the dehydration procedure, at a pressure of 1200 bar. Subsequently, all samples were dried using CO_2_ critical-point drying, covered with palladium gold (Emitech K550 sputter coater, Las Vegas, Nevada, USA and observed via SEM using a Zeiss DSM 940 (Zeiss, Rome, Italy).

### 2.3. Low-Salt Packaging Trials with Table Olives Processed under spCO_2_ Conditions

Olives of the Leccino cultivar subjected to 12 months of fermentation under spCO_2_ conditions were removed from the demijohn bottles and distributed into glass jars. The olives had been marinated for 12 months in brines with 6% and 11% (*w v*^−1^) NaCl and 0.3% (*w v*^−1^) citric acid, both enriched with *S. cerevisiae*. The olives were first carefully washed with water and then divided into glass jars equipped with a metal lid with a “click” vacuum indicator device. Each jar contained approximately 380 g of olives covered with approximately 280 mL of fresh brine with 4% (*w v*^−1^) NaCl and 0.3% (*v v*^−1^) lactic acid. After filling, the jars were hermetically sealed, pasteurized at 82 °C for 10 min, and finally stored in the dark at room temperature. After 6 months of storage, three jars for each type of treatment were subjected to physical–chemical and sensorial analyses. The sensory analysis of the fruits, and the NaCl and total polar phenol content of the olives were evaluated according to the methods described above, whereas the olive pulp biophenols were determined through high-performance liquid chromatography (HPLC) analysis.

#### 2.3.1. Olive Pulp Biophenol Analysis

##### Preparation of Phenolic Extract

Phenolic extract from olives was prepared with the finely chopped pulp of 30 randomly selected fruits. After slight mixing, 2 g was weighed out, and 1 mL of internal standard was added (syringic acid 0.015 mg mL^−1^ in methanol/water, 80:20). After 30 s of stirring, 5 mL of methanol/water mixture (80:20, *v v*^−1^) was added and stirred again for 1 min. The extraction of the phenolic compounds was completed through bath ultrasonication (Ultrasonic Cleaner AU 32, Argo Lab, Arezzo, Italy) for 15 min and centrifugation at 5000× *g* for 25 min (Hettich Instruments). The recovered supernatant was microfiltered through a nitrocellulose filter with porosity of 0.45 µm (Minisart NML-Sartorius, Göttingen, Germany) and then used for HPLC analysis.

##### HPLC Analysis

HPLC analysis was performed on a Jasco LC-4000 liquid chromatographic system equipped with a DAD detector (Jasco Europe s.r.l., Cremella, Italy). A Nucleosil C18 column (4.60 mm i.d. × 250 mm; particle size 5 µm) (Phenomenex, Torrance, CA90501-1430, USA) was used. The injected sample volume was 20 µL, and the elution was performed at a flow rate of 1.0 mL min^−1^, with water and 0.2% phosphoric acid as solvent A, methanol as solvent B, and acetonitrile as solvent C. The elution gradient was as follows: at 0 min, solvent ratio of 96% A, 2% B, and 2% C; at 40 min, solvent ratio of 50% A, 25% B, and 25% C; at 45 min, solvent ratio of 40% of A, 30% B, and 30% C; at 60 min, solvent ratio of 0% of A, 50% B, and 50% C; at 70 min, solvent ratio of 0% A, 50% B, and 50% C; at 72 min, solvent ratio of 96% A, 2% B, and 2% C, maintained for 10 min. The total analysis time was 82 min. Chromatograms were acquired at wavelengths of 200 and 400 nm. Compound identification and quantification were performed by consideration of the retention time and absorption at different wavelengths. The biophenol content is expressed as mg tyrosol equivalent kg^−1^ olive pulp. The analysis was repeated three times for each olive sample.

### 2.4. Statistical Analysis

Statistical software was used for data processing (Statsoft version 7.0 for Windows, Tulsa, OK, USA). Comparisons among means were performed using Duncan’s multiple-range tests (one-way ANOVA), and differences were considered significant at *p* < 0.05. 

## 3. Results and Discussion

### 3.1. Incubation Tests in Anaerobiosis and spCO_2_

The traditional natural processing of black table olives in barrels with brine with a high NaCl content involves several critical issues that could be resolved through fermentation in the presence of spCO_2_ self-produced through the respiration of the fruits and the fermentation process. Fermentation under spCO_2_ occurs in hermetically closed containers; therefore, in this process, unlike the traditional process, the fermenters do not need to be refilled with new brine during fermentation, the development of harmful microbial biofilms on the brine is prevented, and anaerobic conditions are ensured in all barrels. Consequently, the costs associated with the management of multiple stacked barrels, and the processing of waste due to spoilage, are decreased. Applying the “hurdles concept” to the natural processing of table olives, we added spCO_2_ as a third means of preventing microbial growth, thus increasing the antimicrobial activity at low pH, and decreasing the NaCl concentration to 6% (*w v*^−1^) beyond that achieved under the low pH and high NaCl concentration of 11% (*w v*^−1^) typically used in the brine of naturally processed black table olives. Brine stored in hermetically sealed demijohn bottles generated a light pressure of 0.5 bar during the first 24 h of incubation, and subsequently reached 2 bar and remained constant throughout the fermentation. Microbiological analysis of Leccino table olives processed with 6% (*w v*^−1^) NaCl and 0.5% (*w v*^−1^) citric acid indicated an absence of bacteria and molds in brine subjected to spCO_2_ after 1 month of incubation, in contrast to the control conditions. In the following months, no substantial differences in microbial growth were observed with respect to the control without spCO_2_ (Table 1). The number of yeasts in the control without spCO_2_ increased during the first 3 months and stabilized in subsequent months. However, in the brine of olives processed in spCO_2_ conditions, the yeast concentration was very low in the first 3 months and subsequently increased without fully reaching control levels. The results (Table 1) demonstrated that fermentation of the olives under spCO_2_ conditions, compared with control conditions, occurred in the absence of bacteria and molds and in the presence of a low number of yeasts. The lowest values of total yeasts in the brine subjected to spCO_2_, compared with the control, were recorded in the first 3 months of incubation, and maximum decreases of 60–90% were observed. The number of yeasts in the brine with spCO_2_ decreased to 33% of that of the control at the 6th month and to 10% of that of the control in subsequent months of incubation (Table 1). The results are in agreement with findings from other pilot studies investigating the use of spCO_2_ to enhance brine antimicrobial activity, in which the total number of yeasts was below the detection limit of the method during the first months of incubation of the olives in brine [15]. The slow increase in the number of total yeasts recorded after the third month of incubation in brine subjected to spCO_2_ is attributable to the differing resistance of microbial cells to adverse conditions through the use of baroprotective compounds [26]. Microbiological analyses performed on the yeast species present in the brines indicated a clear prevalence of species such as *Candida boidinii* and *Candida manshurica* in the control at the beginning and at the end of the incubation period. However, when the brines were subjected to spCO_2_, more species were present in the first 6 months of fermentation, and the prevalence of *S. cerevisiae* reached 60% after 6 months of incubation (Table 1).

These results are consistent with those from the studies cited above, thus confirming the ability of spCO_2_ to inhibit the growth of some oxidizing yeasts, and to foster oleuropeinolytic and fermenting *S. cerevisiae*. More generally, spCO_2_ appears to control microbial growth in olive brines during the first months of fermentation by decreasing the metabolic activity of oxidizing yeasts active on citric acid and other organic acids, which often cause product spoilage associated with elevated pH. The physicochemical analyses confirmed the different metabolic activity in the brines subjected to spCO_2_ compared with the control. In fact, excluding the slight acidifying effect of CO_2_, the titratable acidity present in the brines subjected to spCO_2_ after 1 year of incubation was very similar to the control levels of 0.5% (Table 2). 

Similarly, the higher content of total polar phenols indicated a lower catabolic activity in the brine subjected to spCO_2_ than in the control, in which many phenolic compounds were degraded. However, the greater accumulation of phenolic compounds in the olive pulp treated with spCO_2_ did not negatively affect the debittering process, as evidenced by the bitterness index (K_225_) and sensory score attributes (Table 2 and Table 3). 

Sensory analysis indicated a lower overall quality score in the control olives, mainly because of anomalous fermentation and rancidity. These results correlated with the high concentrations of yeasts found in the control brine during the entire incubation period (Table 1). The excessive growth of yeasts usually results in a final product with milder taste and less self-preservation [27]. In contrast, the predominant oxidizing yeasts in the brine might have acted on the triglycerides in the fruits through the excessive production of enzymes.

### 3.2. Table Olive Processing Tests with spCO_2_

Table olive processing tests with spCO_2_ allowed us to study the effects of factors such as salt concentration, titratable acidity of the brine, and the use of *S. cerevisiae* inoculation on fermentation progress. The inhibitory effect of NaCl on microbial growth, particularly toward yeasts, varied by the salt concentration and the titratable acidity of the brine. The presence of bacteria and fungi was not observed in any type of brine during incubation. The tests performed with brine with a low salt content of 6% (*w v*^−1^) NaCl without inoculation with *S. cerevisiae* indicated a high number of total yeasts in brine samples with the 0.3% (*w v*^−1^) citric acid after 12 months of incubation. Furthermore, in the same brines containing 0.6% (*w v*^−1^), slightly slowed yeast growth was observed in the first 3 months of incubation. However, these differences due to different titratable acidity were not confirmed in the same brines inoculated with *S. cerevisiae* (Table 4). 

Other tests performed with brines not inoculated with *S. cerevisiae* under high salt conditions at 11% (*w v*^−1^) NaCl, with 12 months of incubation, showed a gradual increase in the total number of yeasts during the first 6 months in the samples of brine enriched with 0.3% (*w v*^−1^) citric acid. In contrast, in the same brines with 0.6% (*w v*^−1^) citric acid, yeasts appeared consistently only after the first 6 months of incubation. Inoculation with *S. cerevisiae* alleviated the negative effects of 0.3% (*w v*^−1^) citric acid, whereas 0.6% (*w v*^−1^) citric acid led to a lower number of yeasts in the brines during the entire incubation period (Table 5). Processing olives under spCO_2_ favored the predominance of *S. cerevisiae* in all types of brines. In non-inoculated brines, *S. cerevisiae* had a prevalence between 55% and 90%. The other species, found in different proportions, were *C. boidinii*, *Wickerhamomyces anomalus*, and *Zygotorulaspora mrakii*. In brines inoculated with *S. cerevisiae*, throughout the incubation period, this yeast species reached 100% in all samples, regardless of the salt concentration and titratable acidity of the brine (Table 4 and Table 5).

**Figure 1:**
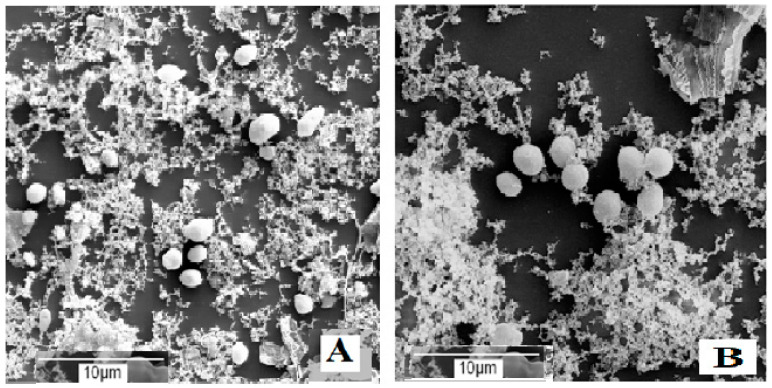
Microbial development in brine not inoculated with *S. cerevisiae*, containing 11% NaCl plus 0.3% (**A**) or 0.6% (**B**) citric acid, after 6 months of incubation.

These results were consistent with the data reported in Table 1, thus confirming that in the presence of spCO_2_, the salt and citric acid concentration of the brine strongly control the growth microorganisms present in the brines. According to SEM observations in brines not inoculated with *S. cerevisiae* and containing 11% (*w v*^−1^) NaCl, after 6 months of incubation, bacteria and fungi were absent. In contrast, the number of yeasts in brines with 0.6% (*w v*^−1^) citric acid was substantially lower than that observed in brine with 0.3% (*w v*^−1^) citric acid (Figure 1A,B), and these microorganisms were below the detection limit of 10 CFU mL^−1^ in the microbiological analysis reported in Table 5.

The antimicrobial action of the brines described above was also observed in brines inoculated with *S. cerevisiae*. In this case, however, it appears less evident because with the inoculation of the brines, we initially started with a high microbial concentration of fermenting yeast *S. cerevisiae*, which, as reported above, had a greater prevalence and therefore greater spCO_2_ tolerance than other species (Table 5). The physicochemical characteristics of Leccino olives processed for 12 months under spCO_2_ with brines with low and high salt contents and different citric acid concentrations did not show significant variations (Table 6). The pH values remained close to the hygienic safety threshold in all samples analyzed [25]. The titratable acidity was not influenced by the salt concentration and varied according to the concentrations of citric acid initially added to the brine. The concentration of NaCl in the olive pulp was lower than that of the brine, thus demonstrating that after 12 months of incubation, the osmotic balance between the brine and the olive pulp had not yet been fully reached. However, the olive pulp processed under spCO_2_ in brines with 11% (*w v*^−1^) NaCl showed a salt concentration ranging from 7.01% to 7.72% at the end of incubation. In contrast, in the pulp of the same olives processed in brines with 6% (*w v*^−1^) NaCl, the salt concentration was significantly lower, ranging from 3.43% to 4.09%. In all samples, no changes were observed in total polar phenols (Table 6).

### 3.3. Low-Salt Packaging Tests

Low-salt packaging tests were performed with the above table olives inoculated with *S. cerevisiae* processed under spCO_2_ in low-salt brines with 6% NaCl or with 11% NaCl and 0.3% citric acid. The physicochemical analyses of the two types of olives, packaged in jars with brine containing 4% *(w v*^−1^) NaCl and 0.3% (*w v*^−1^) lactic acid, after 6 months of storage, indicated a substantial difference in salt concentration in the olive pulp equal to 45%. In detail, olives previously processed in brine with 11% (*w v*^−1^) NaCl, after packaging for 6 months, showed 6.80% (*w v*^−1^) NaCl in olive pulp, while in the olive previously processed in brine with 6% (*w v*^−1^) of salt, 3.75% (*w v*^−1^) NaCl was detected in the pulp. These values, referring to the whole fruit, corresponded to 4.76% (*w w*^−1^) NaCl and 2.63% (*w w*^−1^) NaCl, respectively (Table 7). The substantial decrease in NaCl observed in olives packaged after processing under spCO_2_ with 6% (*w v*^−1^) NaCl was primarily attributable to the low concentration of NaCl at the end of fermentation or at the time of packaging (approximately 4.09% in the pulp; Table 7). In contrast, in the same olives processed with the commonly used high concentration of NaCl of 11% (*w v*^−1^), the NaCl content in the pulp at the time of packaging reached 7.72% (*w v*^−1^) NaCl and therefore was too high to be reduced beyond certain limits in fresh brine with 4% (*w v*^−1^) NaCl present in the jars (Table 7). The olives packaged in glass jars with brine at 4% (*w v*^−1^) NaCl and 0.3% (*w v*^−1^) lactic acid, after 6 months of storage, showed a decrease in NaCl concentration of 8% and 25%, depending on whether they were previously processed in the presence of 6% (*w v*^−1^) or of 11% (*w v*^−1^) NaCl, respectively (Table 7). However, although olives processed with the traditional NaCl concentration of 11% (*w v*^−1^) showed a greater NaCl decrease, after 6 months of packaging, they had 45% more NaCl than olives processed with less salt. The total polar phenol content in the olive pulp processed under spCO_2_ with 6% (*w v*^−1^) and 11% (*w v*^−1^) NaCl decreased 25% and 33%, respectively, 6 months after packaging, and stabilized at 1.71–1.78 mg CAE g^−1^ pulp. The individual biophenols migrated from the pulp to the brine in the jars at different rates depending on the compound, thereby slightly influencing the flavor of the fruits (Table 7).

The results of the sensory scores of olives stored for 6 months in glass jars showed substantial differences for only several attributes: packaged olives previously processed under spCO_2_ with 6% (*w v*^−1^) NaCl had higher scores than the same olives processed with 11% (*w v*^−1^) NaCl, particularly regarding salty (Table 8).

The bitter taste was also slightly more marked in the olives processed with a high NaCl content, probably because of the higher content of bitter glycosides such as oleuropein and its aglycons (Table 7).

## 4. Conclusions

The natural processing of black table olives is increasingly being used, because this method is inexpensive and environmentally friendly. However, several critical issues hinder process management and the quality of the finished product, owing to the refilling of evaporated brines, the growth of biofilms with spoilage yeast on the brines, and the high salt content in the olive pulp. Fermentation in the presence of spCO_2_ is a new olive processing system that, in contrast to the traditional method, can control the growth of spoilage microorganisms in brine with a low salt content. Fermentation under spCO_2_ with 6% (*w v*^−1^) NaCl meets the IOC indications and simultaneously allows the salt concentration in packaged olives to be decreased by 45%. Subsequent packaging and pasteurization of the product in low-salt brine acidified with lactic acid enables a further decrease in salt content and results in a good shelf life of the finished product. Further studies are underway in which this new processing system is being applied on an industrial scale with 160–200 kg barrels.

## Figures and Tables

**Table 1:** Microbial growth in brines of naturally processed Leccino table olives with 6% (*w v*^−1^) NaCl and 0.5% (*w v*^−1^) citric acid during 1 year of incubation under anaerobiosis and slightly pressurized CO_2_ (spCO_2_) conditions.

Month	Total Yeasts (Log CFU mL^−1^)			Preeminent Yeast Species (%)		Total Bacteria (Log CFU mL^−1^)		Total Molds (Log CFU mL^−1^)	
	Anaerobiosis	SpCO_2_	∆ (%) ^1^	Anaerobiosis	SpCO_2_	Anaerobiosis	SpCO_2_	Anaerobiosis	SpCO_2_
1	4.67 ± 0.12 ^a^	1.85 ± 0.21 ^b^	−60	*C.b.* (100)	*S.c.* (63) *C.b.* (27) *W.a.* (8) *G.a.* (2)	1.06 ± 0.50	0 ^2^	1.50 ± 0.10	0
3	6.73 ± 0.02 ^a^	0.70 ± 0.01 ^b^	−90	*C.b.* (94) *C.d.* (6)	*S.c.* (50) *C.b.* (30) *W.a.* (20)	0	0	0	0
6	6.43 ± 0.11 ^a^	4.29 ± 0.16 ^b^	−33	*C.b.* (95) Others (5)	*S.c.* (55) *C.b.* (25) *N.m.*(20)	0	0	0	0
9	5.80 ± 0.11 ^a^	5.23 ± 0.20 ^a^	−10	*P.m.* (92) Others (8)	*S.c.* (92) Others (8)	0	0	0	0
12	5.90 ± 0.15 ^a^	5.02 ± 0.10 ^a^	−15	*P.m.* (80) Others (20)	*S.c.* (90) Others (10)	0	0	0	0

^1^ Percentage of total yeasts grown in slightly pressurized CO_2_ compared with anaerobiosis (control). ^2^ Below the detection limit of 10 CFU mL^−1^. *C.b.*, *Candida boidinii*; *S.c.*, *Saccharomyces cerevisiae*; *W.a.*, *Wickerhamomyces anomalus*; *G.a.*, *Groenewaldozyma auringiensis*; *C.d.*, *Candida diddensiae*; *N.m.*, *Nakazawaea molendinolei*; *P.m.*, *Pichia manshurica*. Mean ± standard deviation (n. repetitions = 3). Values in lines with different letters are significantly different from each other at *p* < 0.05.

**Table 2:** Physicochemical characteristics of naturally processed Leccino table olives with 6% (*w v*^−1^) NaCl and 0.5% (*w v*^−1^) citric acid, after 1 year of incubation under anaerobiosis and slightly pressurized CO_2_ (spCO_2_) conditions.

Parameters	Anaerobiosis		SpCO_2_	
	Olive Brine	Olive Pulp	Olive Brine	Olive Pulp
pH	4.27 ± 0.13	n.d. ^1^	4.30 ± 0.23	n.d.
Acidity (g citric acid L^−1^)	3.58 ± 0.28 ^b^	n.d.	4.45 ± 0.40 ^a^	n.d.
Acidity (g lactic acid L^−1^)	4.63 ± 0.18 ^b^	n.d.	5.75 ± 0.20 ^a^	n.d.
NaCl (%, *w v*^−1^)	4.70 ± 0.13	4.50 ± 0.25	4.19 ± 0.15	4.00 ± 0.10
Bitterness (K_225_)	n.d.	2.51 ± 0.40	n.d.	2.54 ± 0.54
Total polar phenols ^2^ (mg CAE g^−1^)	1.72 ± 0.09 ^c^	2.01 ± 0.05 ^b^	2.38 ± 0.11 ^a^	2.41 ± 0.06 ^a^

^1^ n.d., not detected. ^2^ CAE, caffeic acid equivalent. Mean ± standard deviation (n. repetitions = 3). Values in lines with different letters are significantly different from each other at *p* < 0.05.

**Table 3:** Sensory score attributes of naturally processed Leccino table olives with 6% (*w v*^−1^) NaCl and 0.5% (*w v*^−1^) citric acid after 1 year of incubation under anaerobiosis and slightly pressurized CO_2_ (spCO_2_) conditions.

Gustatory and Olfactory Attributes	Anaerobiosis ^1^	SpCO_2_
Acid	3	3
Salty	3	3
Bitter	4	4
Abnormal fermentation	2	4
Musty	3	3
Rancid	2	3
Overall quality	3	4

^1^ Unsatisfactory: 1 point; moderate: 2 points; good: 3 points; excellent: 4 points.

**Table 4:** Effects of yeast inoculation and low salt concentration (6%, *w v*^−1^) of brine with different citric acid concentrations on microbial growth during fermentation of Leccino table olives under CO_2_.

Month	UNINOCULATED						INOCULATED					
	Citric Acid ^1^						Citric Acid					
	0.3%			0.6%			0.3%			0.6%		
	Total Yeasts ^2^	Predominant Yeast Species	Others ^3^	Total Yeasts	Predominant Yeast Species	Others	Total Yeasts	Predominant Yeast Species	Others	Total Yeasts	Predominant Yeast Species	Others
3	5.78 ± 0.10 ^a^	*S.c.* (76) ^4^ *C.b.* (24)	0 ^5^	3.10 ± 0.09 ^b^	*S.c.* (60) *C.b.* (30) Others (10)	0	5.04 ± 0.23 ^a^	*S.c.* (100)	0	5.47 ± 0.09 ^a^	*S.c.* (100)	0
6	5.74 ± 0.20 ^a^	*S.c.* (60) *C.b.* (30) Others (10)	0	4.80 ± 0.15 ^b^	*S.c.* (70) *C.b.* (20) *W.a.* (10)	0	5.73 ± 0.17 ^a^	*S.c.* (100)	0	5.68 ± 0.15 ^a^	*S.c.* (100)	0
9	6.21 ± 0.18	*S.c.* (70) *W.a.* (15) Others (15)	0	5.50 ± 0.21	*S.c.* (60) *W.a.* (20) Others (20)	0	6.42 ± 0.13	*S.c.* (100)	0	5.89 ± 0.19	*S.c.* (100)	0
12	5.60 ± 0.13	*S.c.* (92) *Z.m.* (8)	0	5.04 ± 0.06	*S.c.* (90) Others (10)	0	5.70 ± 0.15	*S.c.* (100)	0	6.06 ± 0.09	*S.c.* (100)	0

^1^ Concentration (*w v*^−1^) of citric acid added to the brine. ^2^ Yeast concentration expressed as Log CFU mL^−1^. ^3^ Total bacteria and molds. ^4^ Percentage dominance. *S.c.*, *Saccharomyces cerevisiae*; *C.b.*, *Candida boidinii*; *W.a.*, *Wickerhamomyces anomalus*; *Z.m.*, *Zygotorulaspora mrakii*. ^5^ Below the detection limit of 10 CFU mL^−1^. Mean ± standard deviation (n. repetitions = 3). Values in lines with different letters are significantly different from each other at *p* < 0.05.

**Table 5:** Effects of yeast inoculation and high salt concentration (11%, *w v*^−1^) in brine with different citric acid concentrations on microbial growth during fermentation of Leccino table olives under slightly pressurized CO_2_.

Month	UNINOCULATED						INOCULATED					
	Citric Acid ^1^						Citric Acid					
	0.3%			0.6%			0.3%			0.6%		
	Total Yeasts ^2^	Predominant Yeast Species	Others ^3^	Total Yeasts	Predominant Yeast Species	Others	Total Yeasts	Predominant Yeast Species	Others	Total Yeasts	Predominant Yeast Species	Others
3	4.05 ± 0.18 ^b^	*S.c.* (70) ^4^ *C.b.* (20) Others (10)	0 ^5^	0	-	0	5.20 ± 0.08 ^a^	*S.c.* (100)	0	3.01 ± 0.20 ^c^	*S.c.* (100)	0
6	6.02 ± 0.20 ^a^	*S.c.* (70) *C.b.* (30)	0	0	-	0	5.60 ± 0.12 ^a^	*S.c.* (100)	0	3.90 ± 0.35 ^b^	*S.c.* (100)	0
9	6.19 ± 0.23 ^a^	*S.c.* (80) *Z.m.* (20)	0	3.50 ± 0.10 ^b^	*S.c.* (60) *C.b.* (30) Others (10)	0	5.93 ± 0.05 ^a^	*S.c.* (100)	0	4.06 ± 0.10 ^b^	*S.c.* (100)	0
12	5.20 ± 0.30 ^a^	*S.c.* (80) *Z.m.* (15) *C.b.* (5)	0	4.50 ± 0.12 ^b^	*S.c.* (55) *Z.m.* (35) *C.b.* (10)	0	5.82 ± 0.07 ^a^	*S.c.* (100)	0	4.62 ± 0.10 ^b^	*S.c.* (100)	0

^1^ Concentration (*w v*^−1^) of citric acid added to the brine. ^2^ Yeast concentration expressed as Log CFU mL^−1^. ^3^ Total bacteria and molds. ^4^ Percentage of dominance. *S.c.*, *Saccharomyces cerevisiae*; *C.b.*, *Candida boidinii*; *Z.m.*, *Zygotorulaspora mrakii*. ^5^ Below the detection limit of 10 CFU mL^−1^. Mean ± standard deviation (n. repetitions = 3). Values in lines with different letters are significantly different from each other at *p* < 0.05.

**Table 6:** Physicochemical characteristics of Leccino table olives after 1 year of fermentation under slightly pressurized CO_2_.

Parameters	NaCl 6%				NaCl 11%			
	UNINOCULATED		INOCULATED		UNINOCULATED		INOCULATED	
	Citric Acid ^1^		Citric Acid		Citric Acid		Citric Acid	
	0.3%	0.6%	0.3%	0.6%	0.3%	0.6%	0.3%	0.6%
Brine pH	4.39 ± 0.33	4.44 ± 0.15	4.32 ± 0.28	4.39 ± 0.18	4.36 ± 0.26	4.35 ± 0.22	4.31 ± 0.31	4.38 ± 0.11
Brine acidity (g citric acid L^−1^)	3.05 ± 0.37 ^b^	5.29 ± 0.31 ^a^	3.55 ± 0.23 ^ab^	5.80 ± 0.29 ^a^	3.98 ± 0.39 ^ab^	5.10 ± 0.12 ^a^	3.52 ± 0.19 ^ab^	6.30 ± 0.05 ^a^
Brine NaCl (%)	5.04 ± 0.28 ^b^	4.71 ± 0.24 ^b^	4.97 ± 0.20 ^b^	4.74 ± 0.21 ^b^	8.19 ± 0.41 ^a^	7.90 ± 0.23 ^a^	9.01 ± 0.36 ^a^	7.70 ± 0.47 ^a^
Olive pulp NaCl (%)	4.09 ± 0.10 ^b^	3.82 ± 0.15 ^b^	3.60 ± 0.12 ^b^	3.43 ± 0.22 ^b^	7.72 ± 0.20 ^a^	7.45 ± 0.10 ^a^	7.60 ± 0.15 ^a^	7.01 ± 0.21 ^a^
Brine total polar phenols (mg CAE mL^−1^)	2.01 ± 0.10	2.07 ± 0.05	2.04 ± 0.05	2.02 ± 0.01	2.10 ± 0.16	2.11 ± 0.04	2.02 ± 0.08	2.15 ± 0.01
Olive total polar phenols (mg CAE g^−1^ pulp)	2.28 ± 0.06	2.30 ± 0.01	2.35 ± 0.07	2.38 ± 0.05	2.64 ± 0.01	2.70 ± 0.04	2.70 ± 0.03	2.75 ± 0.02

^1^ Concentration (*w v*^−1^) of citric acid added to the brine. CAE, caffeic acid equivalent. Mean ± standard deviation (n. repetitions = 3). Values in lines with different letters are significantly different from each other at *p* < 0.05.

**Table 7:** Concentrations of salt and phenolic compounds in the pulp and brine of Leccino olives processed for 1 year under spCO_2_ and packaged in jars for 6 months in brine with 4% NaCl and 0.3%(*v v*^−1^) lactic acid.

Parameters	NaCl 6% (*w v^−^*^1^)			NaCl 11% (*w v^−^*^1^)		
	Original	Packaged	∆ (%) ^1^	Original	Packaged	∆ (%)
Brine NaCl (%, *w v^−^*^1^)	5.04 ± 0.28 ^b^	4.04 ± 0.07 ^c^	−20	8.19 ± 0.41 ^a^	4.23 ± 0.11 ^c^	−48
Pulp olive NaCl (%, *w w^−^*^1^)	4.09 ± 0.02 ^c^	3.75 ± 0.27 ^c^	−8	7.72 ± 0.20 ^a^	6.80 ± 0.36 ^b^	−12
Whole olive NaCl (%, *w w^−^*^1^)	2.86 ± 0.12 ^b^	2.63 ± 0.15 ^b^	−8	5.40 ± 0.22 ^a^	4.76 ± 0.30 ^a^	−12
Pulp olive biophenol ^2^:						
Hydroxytyrosol	218 ± 12 ^ab^	153 ± 9 ^c^	−30	248 ± 10 ^a^	201 ± 8 ^b^	−19
Tyrosol	22 ± 0.56 ^b^	20 ± 0.43 ^b^	−9	30 ± 0.92 ^a^	30 ± 1 ^a^	0
Vanillic acid	9 ± 0.10 ^ab^	8 ± 0.12 ^b^	−11	13 ± 0.88 ^a^	12 ± 0.67 ^ab^	−8
Hydroxytyrosol acetate	0	0	-	17 ± 0.54	17 ± 0.87	0
*p*-Coumaric acid	<1	<1	0	<1	<1	0
Decarboxymethyl oleuropein aglycone, oxidized dialdehyde form	54 ± 9 ^a^	7 ± 0.30 ^c^	−87	57 ± 8 ^a^	29 ± 0.76 ^b^	−49
Decarboxymethyl oleuropein aglycone, dialdehyde form	<1	<1	0	2 ± 0.10	<1	−55
Oleuropein	6 ± 0.11 ^ab^	3 ± 0.09 ^b^	−50	9 ± 0.17 ^a^	8 ± 0.12 ^a^	−11
Oleuropein aglycone, dialdehyde form	<1	<1	0	5 ± 0.06	4 ± 0.03	−20
Tyrosol acetate	19 ± 0.87 ^b^	7 ± 0.10 ^c^	−63	26 ± 1 ^a^	20 ± 0.9 ^ab^	−23
Decarboxymethyl ligstroside aglycone, dialdehyde form	1 ± 0.01	<1	−10	<1	<1	0
Pinoresinol, 1-acetoxy- pinoresinol	3 ± 0.08 ^b^	2 ± 0.01 ^b^	−33	7 ± 0.07 ^a^	7 ± 0.10 ^a^	0
Cinnamic acid	7 ± 0.0 ^a^	1 ± 0.01 ^b^	−86	1 ± 0.00 ^b^	1 ± 0.01 ^b^	0
Oleuropein aglycone, aldehyde and hydroxylic form	17 ± 0.87 ^ab^	15 ± 0.98 ^b^	−12	24 ± 1 ^a^	24 ± 1 ^a^	0
Ligstroside aglycone, oxidised aldehyde and hydroxylic form	3 ± 0.65	3 ± 0.26	0	4 ± 0.06	4 ± 0.15	0
Apigenin	25 ± 2 ^b^	10 ± 1 ^c^	−60	37 ± 0.97 ^a^	37 ± 2.0 ^a^	0
Methyl-luteolin	<1	<1	0	3 ± 0.77	3 ± 0.86	0
Ligstroside aglycone, aldehyde and hydroxylic form	<1	<1	0	<1	<1	0
Pulp olive total polar phenols (mg CAE g^−1^)	2.28 ± 0.06 ^ab^	1.71 ± 0.02 ^b^	−25	2.64 ± 0.01 ^a^	1.78 ± 0.02 ^b^	−33

^1^ Percentage of each parameter determined in the original sample compared with the packaged sample. ^2^ mg kg^−1^ olive pulp. CAE, caffeic acid equivalent. Mean ± standard deviation (n. repetitions = 3). Values in lines with different letters are significantly different from each other at *p* < 0.05.

**Table 8:** Sensory score attributes of naturally processed Leccino olives for 1 year under spCO_2_ and then packaged in jars for 6 months in brine with 4% (*w v*^−1^) NaCl and 0.3% *(v v*^−1^) lactic acid.

Gustatory and Olfactory Attributes	NaCl 6% *(w v^−^*^1^)		NaCl 11% *(w v^−^*^1^)	
	Original ^1^	Packaged	Original	Packaged
Acid	3	3	3	3
Salty	3	4	1	2
Bitter	4	4	3	3
Abnormal fermentation	4	4	4	4
Musty	4	4	4	4
Other defects	4	4	4	4
Overall quality	3	4	1	2

^1^ Unsatisfactory: 1 point; moderate: 2 points; good: 3 points; excellent: 4 points.

## Data Availability

Data is contained within the article.

## References

[1] International Olive Council (IOC) (2022). Economic Affairs & Promotion Unit. World Table Olive Figures. https://www.internationaloliveoil.org/what-we-do/economic-affairs-promotion-unit/#figures.

[2] Değirmencioğlu N., Boskou D., Clodoveo M.K. (2016). Modern techniques in the production of table olives. Products from Olive Tree.

[3] Johnson R.L., Mitchell A.E. (2018). Reducing Phenolics Related to Bitterness Table Olives. J. Food Qual..

[4] Aponte M., Ventorino V., Blaiotta G., Volpe G., Farina V., Avellone G., Lanza C.M., Moschetti G. (2010). Study of green Sicilian table olive fermentations through microbiological, chemical and sensory analyses. Food Microbiol..

[5] Ciafadini G., Zullo B.A. (2020). Use of air-protected headspace to prevent yeast film formation on the brine of Leccino and Taggiasca black table olives processed in industrial-scale plastic barrels. Foods.

[6] Ozdemir Y., Guven E., Ozturk A. (2014). Understanding the characteristics of oleuropein for table olive processing. J. Food Process Technol..

[7] Anagnostopoulos D.A., Tsaltas D. (2021). Current status, recent advances, and main challenges on table olive fermentation: The present meets the future. Front. Microbiol..

[8] World Health Organization (WHO) (2007). Reducing Salt Intake in Populations: Report of a WHO Forum and Technical Meeting.

[9] Chrysant S.G. (2016). Effects of high salt intake on blood pressure and cardiovascular disease. The role of COX inhibitors. Clin. Cardiol..

[10] Us Department of Agriculture and US Department of Health and Human Services (2020). Dietary Guidelines for Americans, 2020–2025. DietaryGuidelines.gov.

[11] European Parliament and Council of the European Union (2011). Regulation (EU) No 1169/2011 of the European Parliament and of the Council of 25 October 2011 on the provision of food information to consumers. Off. Eur. Union.

[12] Penland M., Pawtowski A., Pioli A., Maillard M.B., Debaets S., Deutsch S.M., Coton M. (2022). Brine salt concentration reduction and inoculation with autochthonous consortia: Impact on Protected Designation of Origin Nyons black table olive fermentation. J. Food Res. Int..

[13] Bautista Gallego J., Arroyo Lopez F.N., Romero Gil V., Rodríguez Gómez F., García García P., Garrido Fernández A. (2011). Chloride salt mixture affect Gordal cv. green Spanish-style table olive fermentation. Food Microb..

[14] Zinno P., Guantario B., Perozzi G., Pastore G., Devirgiliis C. (2017). Impact of NaCl reduction on lactic acid bacteria during fermentation of Nocellara del Belice table olives. Food Microb..

[15] Zullo B.A., Ciafardini G. (2022). Use of Slightly Pressurized Carbon Dioxide to Enhance the Antimicrobial Properties of Brines in Naturally Processed Black Table Olives. Microorganisms.

[16] Haas G.J., Prescott H.E., Dudley E., Dik R., Hintlian C., Keane L. (1989). Inactivation of microorganisms by carbon dioxide under pressure. J. Food Saf..

[17] James A.D., Rajagopalan K., Syed S.N.R. (1985). A review of effects of carbon dioxide on microbial growth and food quality. J. Food Prot..

[18] Wei C.I., Balabam M.O., Fernando S.Y., Peplow A.J. (1991). Bacterial effect of high pressure CO_2_ treatment on foods spiked with *Listeria* or *Salmonella*. J. Food Prot..

[19] Yin H., Dong J., Yu J., Chang Z., Qian Z., Liu M., Huang S., Hu X., Liu X., Deng J. (2016). A preliminary study about the influence of high hydrostatic pressure processing on the physic-chemical and sensorial properties of a cloudy wheat beer. J. Inst. Brew..

[20] Huang H.W., Wu S.J., Lu J.K., Shyu Y.T., Wang C.Y. (2017). Current status and future trends of high-pressure processing in food industry. Food Cont..

[21] Heperkan D. (2013). Microbiota of table olive fermentations and criteria of selection for their use as starters. Front. Microbiol..

[22] Zullo B.A., Ciafardini G. (2020). Differential microbial composition of monovarietal and blended extra virgin olive oil determines oil quality during storage. Microorganisms.

[23] Garrido Fernández A., Fernández Diaz M.J., Adams M.R. (1997). Table Olives: Production and Processing.

[24] International Olive Council (IOC) (2021). (IOC/OT) MO No. 1/Rev. 3.

[25] International Olive Council (IOC) (2004). Trade Standard Applying to Table Olives.

[26] Goh E.L.C., Hocking A.D., Stewart C.M., Buckle K.E., Graham H.F. (2007). Baroprotective effect of increased solute concentration on yeasts and moulds during high pressure processing. Inn. Food Sci. Emer. Technol..

[27] Panagou E.Z., Schillinger U., Franz C.M.A.P., Nychas G.J. (2008). Microbiological and biochemical profile of c.v. Conservolea naturally black olives during controlled fermentation with selected strains of lactic acid bacteria. Food Microbiol..

